# Natural Perspective: Mapping Visual Space with Art and Science

**DOI:** 10.3390/vision2020021

**Published:** 2018-05-07

**Authors:** Alistair Burleigh, Robert Pepperell, Nicole Ruta

**Affiliations:** Fovolab, Cardiff School of Art, Cardiff Metropolitan University, Cardiff CF5 2YB, UK

**Keywords:** art, visual space, perspective, natural perspective, geometrical perspective, peripheral visual field

## Abstract

Following its discovery in fifteenth-century Italy, linear perspective has often been hailed as the most accurate method of projecting three-dimensional visual space onto a two-dimensional picture plane. However, when we survey the history of European art it is evident that few artists fully complied with its mathematical rules, despite many of them being rigorously trained in its procedures. In this paper, we will consider how artists have actually depicted visual space, and present evidence that images created according to a “natural” perspective (NP) used by artists are judged as better representations of visual space than those created using standard linear (LP) and curvilinear fisheye (FP) projective geometries. In this study, we built a real three-dimensional scene and produced photographs of the scene in three different perspectives (NP, LP and FP). An online experiment in which we asked people to rank the perspectives in order of preference showed a clear preference for NP compared to the FP and LP. In a second experiment, participants were asked to view the real scene and rate each perspective on a range of psychological variables. Results showed that NP was the most preferred and the most effective in depicting the physical space naturally. We discuss the implications of these results and the advantages and limitations of our approach for studying the global metric and geometrical structure of visual space.

## 1. Introduction

If visual sensation is “how we see the world” then visual space is how we perceive its spatial properties such as object size, shape, and location, that is, its geometrical structure. The exact geometrical structure of visual space remains a subject of interest and debate, and indeed several scientific researchers have argued that it does not have a single, stable structure but several that vary depending on viewing conditions and individual differences [1,2,3].

In this paper, we investigate the structure of visual space and how it can be most effectively depicted in paintings, drawings, photographs, or computer graphics. While vision scientists and psychologists have been studying visual space at least since the time of Hermann von Helmholtz’s researches into physiological optics [4] it could be argued that artists, and indeed architects, have been studying it for far longer. For thousands of years, artists have been faced with the practical problem of how to convey visual experience in a way that is perceptually convincing and aesthetically satisfying to their audience. Moreover, they frequently have little control over how their images will be viewed, under what lighting conditions and from which positions or distances. Yet despite these practical constraints artists have been highly successful in creating images of visual space that are compelling, informative, and memorable, and artworks consistently rank among the most valued objects in our society, both financially and culturally [5].

In this paper, we show how artistic methods of mapping the structure of visual space can complement those used in vision science. By combining knowledge and methods from art and psychology, and by using creative computer technology, we investigate the structure of visual space and how it can be most effectively depicted.

### 1.1. Linear Perspective

In his *Natural History*, Pliny the Elder narrates how Zeuxis, an ancient Greek renowned for his illusionistic skills was able to paint grapes so realistically that they were pecked at by birds. None of Zeuxis’ paintings survive, but we are told he excelled at effects of shading and highlighting that mimicked those seen by when confronting a real visual scene. The idea that realism is achieved by faithfully depicting the optical properties of a scene, that is, the way the light is reflected from surfaces of objects or sources of luminance to the eyes, recurs at several periods of European art history. Ernst Gombrich referred to the development of ever greater optical fidelity in art as “the conquest of reality” and took it to be one of European art’s greatest achievements [5]. This was never truer than during the Renaissance in Italy when artists and architects uncovered the fundamental laws of linear perspective [6]. For the first time, it is argued, artists had at their disposal a systematic and accurate method for converting three-dimensional space to a two-dimensional picture plane in order, as Gombrich puts it, to “render reality as it appeared to the eye” [5]. The method, first used by Fillipo Brunelleschi in the early fifteenth century and described by Leon Battista Alberti in 1435 [7], is mathematically straightforward and can result in compelling depictions of space. We might immediately think of Piero della Francesca’s Flagellation of Christ (1455–60, Urbino, Italy) as an exemplary case.

**Figure 1:**
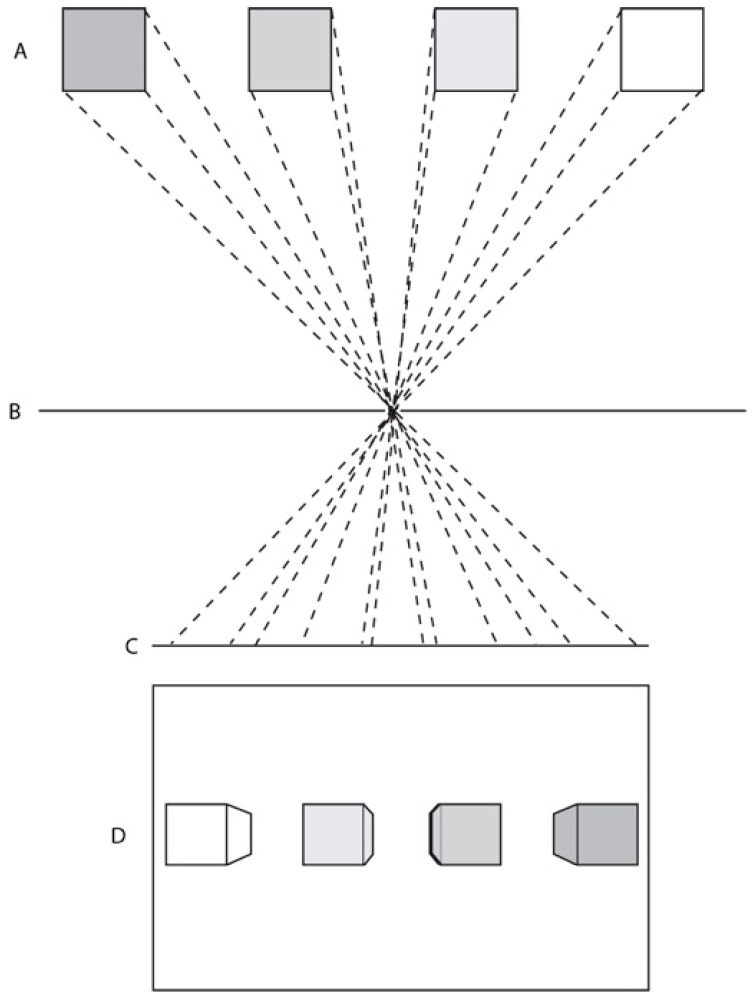
(**A**) schematic diagram of the geometrical principle of linear perspective. Four cubes are shown at (**A**) in plan-view; At (**B**) is shown a light barrier with a centrally located pinhole; (**C**) shows a plane on which the light rays, a subset of which are indicated by dashed lines, travelling from the cubes at A are projected; (**D**) shows an elevation view of the scene as rendered from the projection.

Linear perspective is a geometrical method of modelling the behavior of light as it travels through a small aperture in a barrier to be projected onto a (normally) flat surface. It exploits the fact that light rays travel in straight lines while the aperture permits only a relatively small number of all the available rays in the environment to reach the projection surface (see Figure 1). In the case of a camera obscura or pinhole camera this results in an inverted image on the light receiving plate. Linear perspective geometry can emulate this process using construction lines that converge at a single vanishing point, in the case of one-point perspective, and some simple mathematics to determine the diminution of objects as they recede in pictorial space. Many writers have since argued that linear perspective is the only correct way to depict visual space because, properly arranged and viewed, a linear perspective image will present the single eye with an identical pattern of light to that arriving from the real scene being depicted [8,9,10,11,12,13].

### 1.2. Natural Perspectives

While it has been argued that linear perspective is the only objectively accurate way to depict the visual world over the centuries artists have consistently aware of its limitations [14,15,16]. For example, it cannot effectively capture wide angles of view because objects become increasingly “stretched” with eccentricity as the angle of view increases (the effect seen in photographs taken with wide angle lenses). Consequently, much of the binocular visual field, which extends some 180 degrees horizontally and 130 degrees vertically [17] is usually cropped by the window format of linear perspective, thus excluding much of the peripheral field, including the view we have of our own bodies in that region [6,18,19]. In addition, to view a linear perspective picture properly we need to locate our eye at the exact point of projection in front of the image, since at this point the image projection from the scene coincides with the retinal projection in the eye [20,21]. Unfortunately for most pictures, especially those showing wide-angle views, this point is impractically close to the picture surface and so they tend to appear “stretched” or “distorted” in the margins [11,22]. Nor can linear perspective accommodate the fact we see with two, mobile eyes [23] or the reduction in the apparent size of objects as they recede in the plane perpendicular to the axis of sight [24]. Faced with these limitations, artists rarely applied the laws of linear perspective strictly, and sought other methods of depicting the world that appeared more naturalistic, that is, closer to how they might be expected to appear in natural visual experience [25,26].

In the nineteenth century several alternative methods of depicting reality were proposed based on various forms of non-linear or “natural” perspective [14,27,28]. The term natural perspective gained currency among artists following Leonardo’s usage in respect of depicting objects as they appear to the eye rather than how they would be depicted in “artificial” or mathematical perspective, and therefore differs from the sense employed by Euclid [29]. Natural perspectives tried to accommodate features of visual perception that were difficult or impossible to depict using conventional linear perspective, such as the apparent diminution of objects as they recede from the viewer in the frontal plane and apparent curvature of straight lines in the peripheral visual field [10,19,23,30]. In the twentieth century Albert Flocon and André Barre [24], for example, proposed a mathematical form of curvilinear perspective with five vanishing points in which curvature was defined by segments of ellipses. They claimed this offered a better pictorial approximation to the appearance of visual space in natural vision than linear perspective. An alternative curvilinear perspective proposed by Robert Hansen [31] that attempted to achieve the same aim also used five vanishing points, but hyperbolae rather than ellipses.

Among living artists, David Hockney has been a consistent critic of linear perspective and photography, which he says reduces our experience of mobile, binocular vision to that of a “paralyzed Cyclops” [32]. He has experimented extensively with techniques for depicting visual experience in a way that corresponds more closely to natural visual experience. Most recently he has made a series of paintings and digital collages that attempt to “free us from a chemically imposed perspective” of photography in order to create images that have “an almost 3D effect without the glasses” [33]. In the nineteenth and twentieth centuries several artists experimented with introducing binocular information into pictures by emulating the effects of physiological diplopia, or “double vision”. Notable among these was Blanche Ames [34] who published research on representing the structure of visual space in painting with her brother, the vision scientist Adelbert Ames [35]. The painter Evan Walters [36] made many similar “double-vision” studies in the 1940s [37] and, like, Hockney, referred to the views created by linear perspective as though seen by a Cyclops [36].

Several art historians have shown interest in natural perspectives used by artists. Erle Loran’s [38] study of Paul Cézanne’s compositional methods, based on comparing photographs taken from the same locations as paintings, influenced some mid-twentieth-century artists, and this work was supplemented more recently by Pavel Machotka [39]. In a previous study we showed, using information provided by Loran and Machotka [38,39], that Cézanne tended to use a particular form of pictorial organization that is likely to reflect the perceptual and attentional processes involved when fixating on a central motif in one region of visual space while painting the peripheral space around it [40]. In one example, Cézanne paints a stone well in a forest. When the size of the well in the painting is compared to the linear perspective photograph taken from the same position and showing the same visual space, the well in the painting appears significantly larger than in the photograph, while the surrounding space is relatively compressed in the painting.

This general pattern appears in many artists’ work. For example, Pierre Bonnard, who had a well-documented preoccupation with recording his own visual perception [41,42,43], painted a wide-angle view of the interior of a room. The centrally-viewed radiator in the room appears much larger in the painting than in a photograph of the same scene, while objects in the peripheral foreground are much diminished, following the pattern of pictorial organization favored by Cézanne [44,45]. Michael Baxandall’s [46] close reading of Jean-Baptist-Siméon Chardin’s work, meanwhile, benefits from an understanding of the phenomenology of peripheral vision, and Rod Bantjes [47] has investigated the impact of stereoscopic photography on the representation of depth in nineteenth-century painting. However, little recent art historical work has built on these foundations, and we still lack any systematic or quantitative analysis of how artists have depicted their “visual sensations”—a term frequently used by artists when describing visual space [48,49].

### 1.3. Visual Space

The term visual space refers to the perceived size, distance, and shape of objects in the visual field. It has long been known that visual space does not conform to the 3D Euclidean geometry that describes physical space [36,50]. Helmholtz was the first to scientifically report on the phenomena of perceived curvature, central enlargement, and peripheral compression of the visual field recorded elsewhere by artists. His famous curved checkerboard image illustrates these phenomena [4,10]. The phenomenon of “focal enlargement” of objects under direct fixation was reported in the early twentieth century [51]. However, despite decades of research there still is no general agreement about how visual space is geometrically structured, or indeed if it has any consistent geometrical structure at all. Luneburg [52] proposed a model of visual space that was essentially hyperbolic in structure. Heelan [53] also argued that visual space is hyperbolically curved, and used examples from art, including the work of van Gogh, to support his case. Subsequent experiments have failed to fully support a hyperbolic geometry for visual space, but there is general agreement it is non-Euclidean [3]. The major problem still facing vision science, therefore, is the one identified by Luneburg [52], which is to “establish a metric for the manifold of visual sensations.”

Koenderink and van Doorn [2,54] are among those who argue against there being a universal metric for visual space. Rather they talk about the geometry of visual spaces, which vary depending on factors such as the context in which cues are perceived. In one study, the same authors found the geometry of visual space varied with proximity to the viewer, changing from elliptic in near space to hyperbolic in far space, to parabolic at very large distances [50]. Indow [1] noted that most studies of visual space are concerned with its local properties, while he attempted to give an account of its global structure. Basing his calculations on a range of previous experimental findings he concluded many features of visual space could be modelled using a Riemannian geometry of constant curvature. However, these can only be measured for specific viewing conditions and not generalized for all. Hence our understanding of visual space is, he claims, “pluralistic and fragmentary”. Other authors have agreed [55,56].

The fact that no coherent global metric for visual space has yet been determined may, in part, be because the experimental data depend on sparse measurements made in unnatural viewing conditions. As Wagner [3] acknowledges, most existing studies are unidimensional, that is, they measure either the fronto-parallel plane or the depth plane of vision, but rarely both. Moreover, they are often conducted under conditions we rarely encounter in natural vision, using very minimal stimuli such as dots of light in dark spaces, for example. Indow [1] notes that the fact we usually see with two mobile eyes is hardly ever accounted for. Some researchers have tested more ecologically valid viewing conditions using binocular stimuli in virtual reality environments where observers are able to move their bodies [57]. Here it was found that the expectation that the size of objects in visual space remain constant overrides other spatial cues, such as distance walked or interocular separation. Many studies of visual space are limited by their neglect of the visual far periphery, with judgments generally being made by participants using direct vision only [3,58]. Some theorists have claimed the periphery is irrelevant altogether [59]. Hence little or no data exists about spatial properties of the full binocular visual field [60].

Visual sensation is composed not only of spatial properties but also of “texture” properties such as variations in acuity across the visual field, and the “double-vision” or “diplopic” effects of binocular vision. These contribute to distance and depth judgments in vision [61,62]. Also, visual acuity varies with eccentricity, with the highest resolution coinciding with the foveal area of the retina and falling off rapidly towards the periphery [63]. Although textbooks often characterize the appearance of the visual periphery as “blurry” [64], this is not the case. The lower acuity compared to central vision [17] is likely due to the way the neural activity in the receptive fields is pooled over larger regions with eccentricity rather than the result of defocus [65,66]. Likewise, the phenomenon of physiological diplopia, or “double vision”, has long been recognized as a fundamental feature of natural vision and critical to the perception of depth [67]. Despite this, its potential to function as one of the so-called “monocular depth cues”, which we use to infer depth from pictures, by simulating it in 2D images has barely been exploited because it is widely assumed double vision is “screened out” of conscious vision [68,69].

### 1.4. Mapping Visual Space

**Figure 2:**
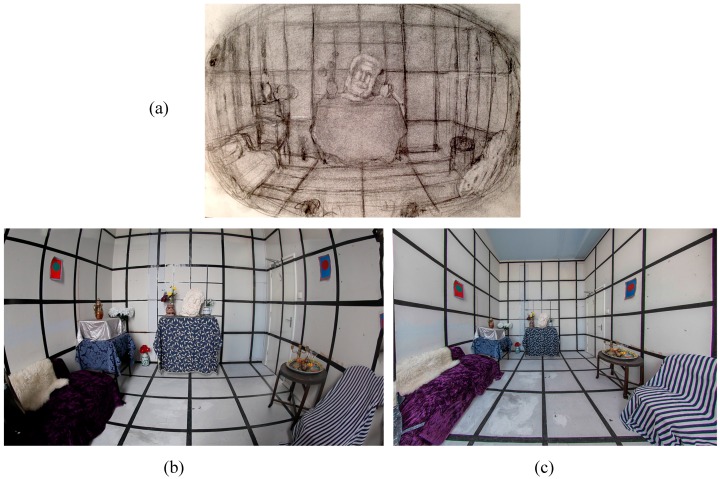
An illustration of the difference between the pictorial organization of an artistic depiction of a three-dimensional scene (**a**) an artistic drawn image (**b**) a fisheye perspective photographic image and (**c**) a linear perspective photographic image of the same scene from the same point of view, centered on the plaster head. Each image depicts approximately the same field of view. The artistic image can be considered a map of the structure of the visual space while the photographs capture in different ways the optical data.

Our research has focused on mapping the geometric structure of the binocular visual field for a given fixation point, including binocular disparity and scaling effects due to depth [70,71]. We define a map of a visual space as a two-dimensional representation, or depiction, of a three-dimensional scene that matches as closely as possible the way that scene is perceived. Figure 2 shows a scene captured using a method of direct empirical observation of the visual field based on a given fixation point (the central plaster head), and how it compares to a photograph from the same viewpoint captured using a fisheye lens (8 mm) and a wide angle rectilinear lens (17 mm). Note the fixated head in the center of the drawing is much larger in the drawing compared to the photographs. The grid lines in the periphery of the drawing are less curved than in the fisheye photograph, which appear unnaturally bent, but slightly more curved than in the linear perspective photograph, which appears unnaturally stretched in the periphery. As part of this research we have developed methods for quantifying how artists have recorded the structure of visual sensation through painting and drawing [19,40,72]. In addition, we have measured the perceived size and shape of objects across the entire visual field [73,74] and as a function of fixation in depth [51]. This was done by showing observers a real space and asking them to judge which images from a selection of four standard geometrical projections and one artistically generated depiction they judged most closely matched their visual experience. We have also established that emulating physiological diplopia in images can act as an effective monocular depth cue [69].

**Figure 3:**
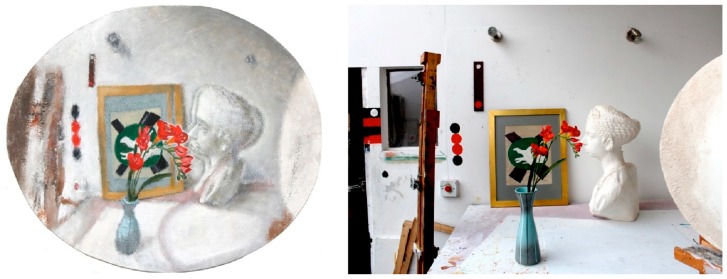
(**Left**) Still life with objects, Robert Pepperell, oil and sand on shaped canvas, 2012. (**Right**) Linear perspective photograph of same physical space depicted in the painting showing approximately the same visual space but taken from a point of view further back than that from which it was painted. Note the relative enlargement of the centrally located objects, the diminution of objects in the periphery of the painting compared to the photograph, as well as the binocular doubling in the area behind the flowers and the fall of in acuity with eccentricity.

The method of mapping visual space generally involves defining a fixation point in the visual world and then visually measuring the perceived sizes, shapes, and positions of all the objects in the space relative to the fixation point [8,75]. Critically, this procedure is carried out without moving the eyes from the fixation point when recording the appearance of peripheral areas of the visual field. In this way the structure of the entire visual field, and the visual space perceived, can be recorded. Figure 3 shows a painting made by one of the authors using this method, and the structure of the depicted visual space can be compared to a linear perspective photograph taken from the same viewpoint. It is also important to note that while the photograph, as with all standard linear perspective projections, is monocular the information captured through the artistic process just described uses binocular information. This is achieved either by “fusing” the two separate binocular images into a single “compound” image, or by emulating the effects of physiological diplopia, or “double vision”, as noted in the work of other artists above.

Our research to date suggests that binocular visual space is structured according to a form of complex non-linear 3D geometry as yet undefined mathematically. The approximate form of this geometry when represented in a two-dimensional image consists in the central area under fixation being generally enlarged compared to the standard geometries, while objects in the periphery are generally compressed [40,72]. Given that this structure is derived from empirical observation of natural human vision rather than the geometry of optics we will refer to this artistically rendered visual space as “natural”, in line with other forms of natural or “perceptual” perspectives previously proposed by artists and others [14,27,28,31,35,44,76,77,78].

For the present study we wished to measure the extent to which a photograph of a three-dimensional space, created according to the natural perspective principles outlined above, would be preferred by observers, or judged to match their own experience of the visual space when compared to more commonly used photographic projections, such as linear perspective and curvilinear fisheye. We were also interested to understand what the natural perspective-generated photograph might reveal about the structure of visual space, and whether the subjective report of the artist about his own visual experience corresponded to that reported by other people when viewing the same space. Our overall hypothesis was that people would prefer the natural perspective photograph to other standard photographs and rate it as being closer to the experience of viewing a real scene.

## 2. Materials and Methods

**Figure 4:**
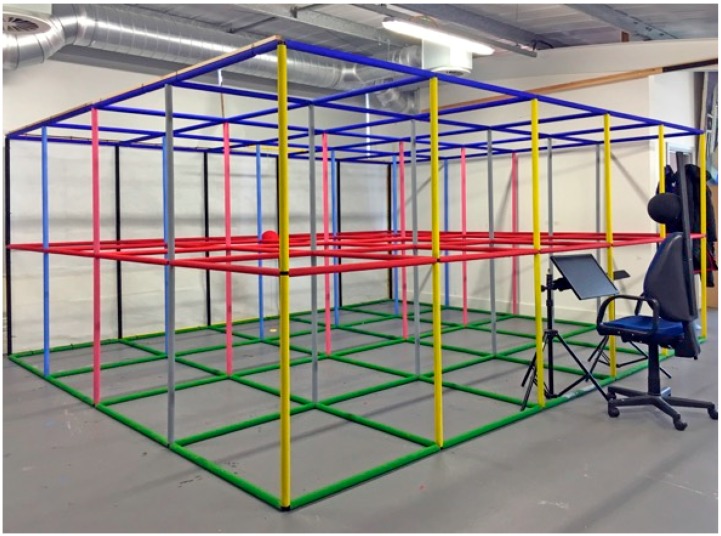
Experimental setup, showing the 3D grid room layout, the chair from which the scene was viewed, the location of the computer monitor, and the fixation sphere.

**Figure 5:**
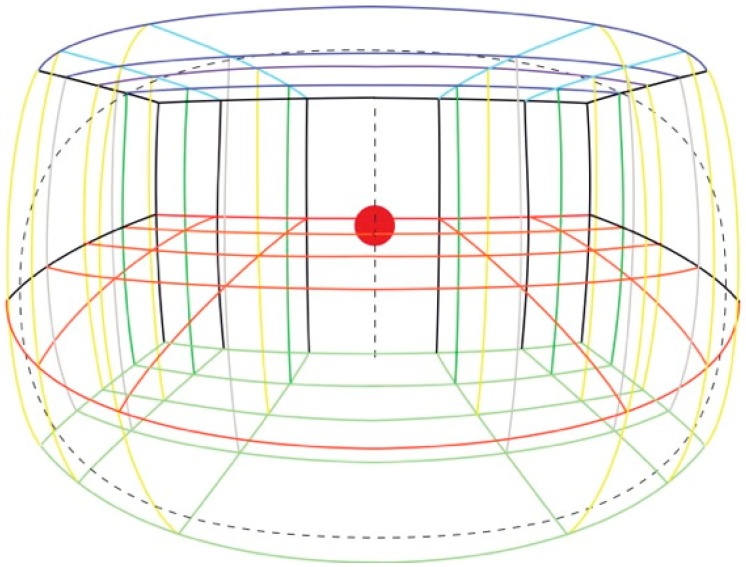
Natural perspective depiction of the 3D grid room. This is a drawing by one of the authors of the subjective visual experience of the experimental space based on a fixation point at the red ball. The drawing is a synthesis of both eye views, and the dotted elliptical line shows the approximate limits of the visual field, as judged from observation by the artist, which in this case is around 150 degrees on the horizontal axis and 90 degrees on the vertical axis.

To carry out our experiments we constructed a three-dimensional visual scene (3D grid room) composed of intersecting metal rods, each 100 cm in length, arranged as a three-dimensional grid of cubes each 100 cm^3^ and stacked in a grid 5 cubes wide by 4 cubes deep by 2 cubes high. See Figure 4. The rods were covered in colored insulation foam, which allowed for easier visual discrimination of the different layers when viewing inside the grid. A polystyrene sphere 20 cm in diameter was suspended within the center of the furthest cube, 350 cm away from the frontal view of the observer, at a height of 100 cm from the ground and subtended 3.27° of visual angle. Using this sphere as the fixation point, one of the authors then made a line drawing of the entire visual space using Adobe Illustrator on a 15-inch Apple Macintosh laptop using the natural perspective principles outlined above. The drawing is shown in Figure 5.

The drawing represented a wide angle of the binocular visual field of the artist, covering approximately 150 degrees of horizontal angle of view, when fixating on the sphere. Previous work has shown that the perceived field of view can vary between individuals [60] and under different viewing condition, being narrower for example when the eyes are converging. The drawing was made from a viewing distance from the screen of 60 cm, which is known to be the average natural accommodation viewing distance for most viewers [79]. We were mindful of the limitations of linear perspective geometry, noted above, which require the viewer of a depiction to adopt the correct viewpoint with respect to the picture plane for optimum viewing experience. These limitations preclude the use of linear perspective for depicting wide-angle scenes (>120 degrees of horizontal angle of view) unless viewing distances to the image are uncomfortably, and indeed impractically, close.

A photographic image of the 3D grid room was made from the same vantage point as that from which the artist made the drawing. The photograph was a 16:9 aspect ratio image shot on a Black Magic Production Camera with a Canon EF—8–15 mm f/4 L NSM fisheye lens to capture a wide field of view. To record as closely as possible the same viewpoint as the artist, the camera was positioned at the same location as the mid-point between the artist’s eyes. Three images were generated from the photograph to use as stimuli, each showing a different perspective of the 3D grid room: Natural Photograph (NP) based on the natural perspective drawing, Linear Photograph (LP) based on linear perspective, and Fisheye Photograph (FP) based on curvilinear perspective. We chose to produce the FP and LP images as comparisons because they are very commonly used in many imaging technologies [80]. The FP was cropped with an elliptical frame to make it more consistent to the NP version, retaining the 180-degree field of view as a feature of this type of photograph. The LP was generated by using the projection conversion software Hugin (http://hugin.sourceforge.net) to convert the original fisheye photograph into a linear photograph with a horizontal field of view of 150 degrees and a vertical field of view of 125 degrees. The LP version was also cropped with an elliptical frame to make the image more consistent to the NP version.

**Figure 6:**
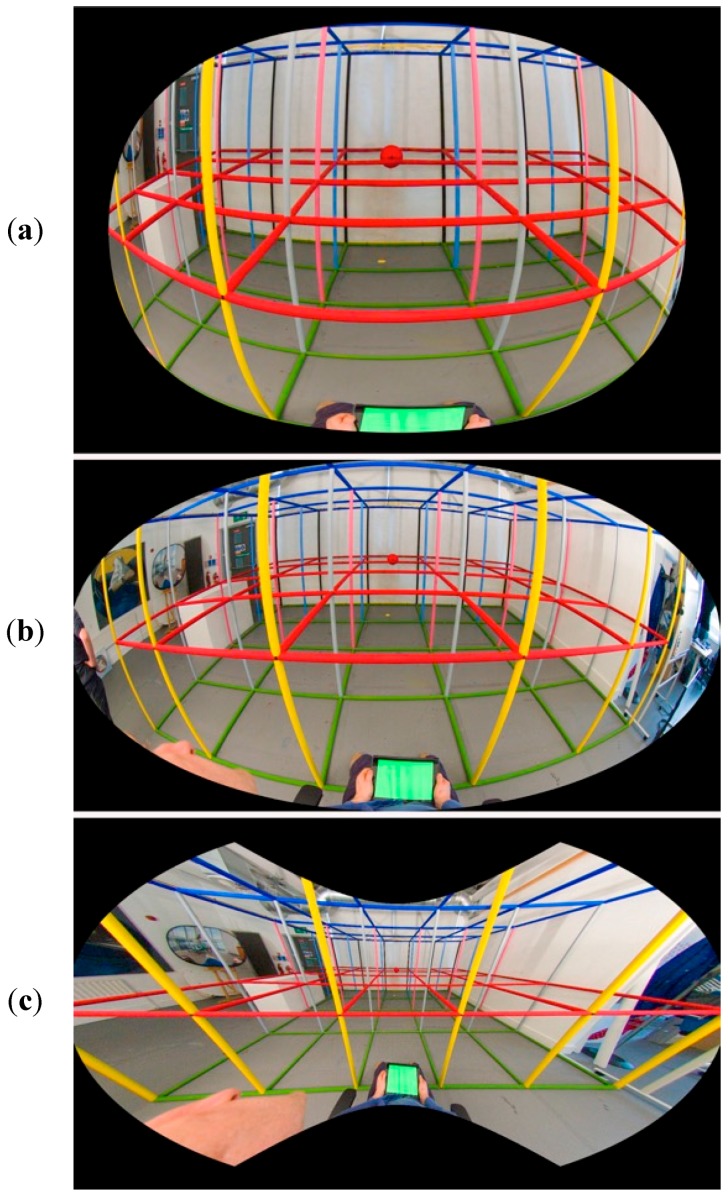
The photographic stimuli used in our experiments, consisting of three different photographs of the same 3D grid room. From top to bottom: (**a**) Natural Photograph (NP), (**b**) Fisheye Photograph (FP) and (**c**) Linear Photograph (LP).

The NP was made by a different method, using the creative digital content production tool Adobe After Effects (www.adobe.com). The FP and drawing were both imported into After Effects and set up as a new composition with the drawing behind the FP photograph in the compositor. The FP photograph was set to 50% transparency to partially reveal the drawing behind and manipulated by one of the authors using the in-built mesh warp modifiers in After Effects to create an image that matched the drawing as closely as possible and by visual reference to the 3D grid room. Figure 6 shows the three stimuli we generated.

## 3. Experiment 1: Online Comparison

Our first experiment was an online study in which participants compared photographs using three different perspectives: Natural Photograph (NP), Fisheye Photograph (FP) and Linear Photograph (LP). The task consisted of ranking the photographs from the most to the least preferred. This was done without participants being able to view the real scene. Given that the FP and LP were depictions of the three-dimensional space that used standard perspectives, and presumably more familiar to people through their use in photography and computer graphics, it would be reasonable to expect them to be preferred to the NP, which may be less familiar. However, based on our previous research we hypothesized that the NP would be preferred for such a wide-angle scene when viewed from a comfortable distance on a standard sized display on the basis that the natural perspective-based NP would compensate for the limitations of standard perspectives under normal viewing conditions.

### 3.1. Participants

Seventy-four participants gave informed consent before taking part to the experiment and all were recruited via the online research tool Prolific (www.prolific.ac), including only those whose main language was English. This was because the experimental instructions were given in English and we wanted to avoid any misunderstanding of the procedure. The average age of participants in this sample was 32.6 years (range 20–49), and 71.6% were female and all had normal or corrected-to-normal vision. Average earnings in our online sample were £3.75 per hour, which were paid via Prolific. To take part to our study, participants had to report to be using a screen bigger than 15 inches (23.38% of participants had 15 inches screen, 44.16% had 17 inches screen and 32.47% had 21 inches screen) and to be sitting on a desk with their computer in front of them at a comfortable distance. Mobile phone and small tablet users were excluded. The experiment was approved by the Ethics Committee of the School of Art and Design, Cardiff Metropolitan University and was conducted in accordance with the Declaration of Helsinki.

### 3.2. Procedure

In this experiment we wanted to investigate if the perspective used to depict the 3D grid room influenced the order in which participants ranked them. The NP, FP and LP stimuli appeared on screen in a random order, arranged in one column. The task was to arrange the photographs from the most to the least preferred while looking at the red ball in the center of the photograph, with the one placed at the top corresponding to the most preferred. Participants could click and drag the pictures to arrange them directly in their preferred order.

### 3.3. Analysis and Results

Descriptive statistics showed that 43.24% of participants assigned the NP to the first position, followed by the FP (36.49%) and the LP (20.27%); 50% of participants assigned the LP to the third position, meaning that half of the time participants choose LP as their least favorite photograph. To determine which was the preferred picture overall, we calculated the weighted ranking score (*R*) for each photograph as follows:

$$R=\;\frac{\left(x_{1}w_{1}+\;x_{2}w_{2}+x_{3}w_{3}\right)}{Total\;frequencies}$$

where *x* is the total frequencies reported by each ranking position and *w* is the weight of the ranked position. The weight was 1 for the photograph given the lowest ranking, 2 for the intermediate ranking and 3 for the highest ranking. As suggested by the percentage of the partial frequencies above, the NP photograph was the most preferred with a score of 2.18, followed by the FP (2.12) and the LP (1.70). A chi-square test of goodness-of-fit was performed to determine whether the three photographs were equally preferred. Preference for the three photographs was not equally distributed in the population: χ^2^ (4, *N* = 74) = 16.621, *p* < 0.05), meaning that there was a significant association between the type of projection used in the photograph and the ranking positions they were assigned to.

Table 1: For each of the three photographs (Natural, Fisheye and Linear), the table shows: in the first raw the absolute percentage of respondents that selected each answer choice; and in the second raw the total frequencies reported for each answer choice. The total responses column reports the total answers collected for each answer choice. The ranking score column lists for each photograph the Total Index of Preference, calculated by summing the weighted ranking for each answer choice.

## 4. Experiment 2: Real Space Comparison

To further investigate and quantify the nature of this difference, we conducted a second experiment in which participants compared the same stimuli, except this time they did so while looking at the real scene being depicted, and fixating on the same object in the scene. We hypothesized that for the given viewing distance and screen size the NP would be preferred to the others, even though the FP and LP were created using standard and hence more familiar forms of perspective.

### 4.1. Participants

Twenty-six participants gave informed consent before taking part to the experiment. The average age of participants in this sample was 29 years (range 21–49), and 38.5% were female and had normal or corrected-to-normal vision. The experiment was approved by the Ethics Committee of the School of Art and Design, Cardiff Metropolitan University and was conducted in the Fovolab facilities in accordance with the Declaration of Helsinki.

### 4.2. Procedure

The experiment was conducted in the Fovolab at Cardiff School of Art and Design, Cardiff Metropolitan University. The experimental apparatus consisted of the 3D grid room space described above, with a height adjustable chair and an Ilyama B2483HS 24 inch 16:9 1080p LED computer display mounted in front of the chair, at a 100 cm height from the floor, such that it could be viewed face on when the participant looked down from 60 cm distance. In this way the display could be referred to without interfering unduly with the visual scene when the participants looked ahead. This setup replicated the conditions under which the drawing of the scene was made, and participants were positioned at this same vantage point, which was also that from which the comparative photographic images were made.

Before starting the experiment, the researcher gave a brief introduction explaining the concept of peripheral vision so that participants understood the requirements of the task. Then, participants were asked to fixate on the red polystyrene ball while paying attention to the whole space in front of them and the full scope of their binocular visual field. The same NP, FP and LP stimuli used in Experiment 1 (Figure 6) were presented individually on screen, with the order counterbalanced between participants. Participants were asked to evaluate each picture on different psychological variables using an agreement 7-point Likert scale, where 1 corresponded to “not at all”, 3 to “neutral” and 7 to “very much”. While expressing their judgments, participants were encouraged to consider their experience of being in and looking at the experimental space, directly comparing it with the photograph on screen.

The task consisted of filling a paper questionnaire measuring preference, spatial presence, ecological validity, and perceptual match. Items measuring spatial presence and ecological validity were selected from the Sense of Presence Inventory [81]. The perceptual match item was customized to better address the purpose of the present study. All items used in our questionnaire and their corresponding psychological variables are reported in Table 2. Sixteen of these participants completed an extended version of the questionnaire, measuring comfort, familiarity, perceived distance of the object in the image, perceived image distortion and how easy it was to fixate on the center of the image. All the additional items used in the extended version of the questionnaire are reported in Table 3.

### 4.3. Analysis and Results

**Figure 7:**
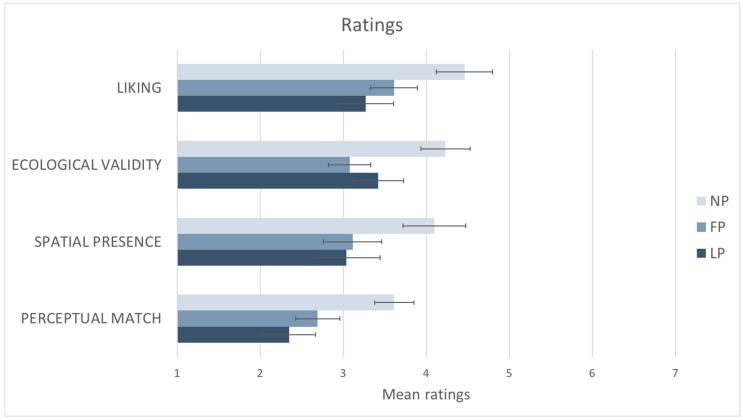
The graph reports the average ratings (*N* = 26) on a 7-point Likert scale for each photograph: Natural (NP), Fisheye (FP) and Linear (LP). Standard errors are represented in the figure by the error bars attached to each column.

Results from the questionnaire are summarized in Figure 7. Results showed that there was a statistically significant difference between liking ratings participants gave to the three different photographs as determined by one-way ANOVA: F (2,50) = 8.770, *p* < 0.001. Post hoc tests using the Bonferroni correction revealed that the mean ratings obtained by the NP (M = 4.46, SD = ±1.2) were statistically significantly higher compared to the ones obtained by the FP (M = 3.61, SD = ±1.36; *p* < 0.025) and the LP (M = 3.26, SD = ±1.63; *p* < 0.025). The NP was judged as having statistically significantly more spatial presence than the other photographs as determined by one-way ANOVA: F (2,50) = 6.982, *p* < 0.05. Post hoc tests using the Bonferroni correction revealed that the mean rating obtained by the NP (M = 4.09, SD = ±1.51) was statistically significantly higher compared to the ones obtained by the FP (M = 3.11, SD = ±1.29; *p* < 0.025) and the LP (M = 3.04, SD = ±1.55; *p* < 0.025).

There was a statistically significant difference between ecological validity ratings participants gave to the three photographs as determined by one-way ANOVA: F (2,50) = 5.861, *p* < 0.025. Post hoc tests using the Bonferroni correction revealed that the mean ratings obtained by the NP (M = 4.23, SD = ±1.92) were statistically significantly higher compared to the ones obtained by the FP (M = 3.07, SD = ±1.78; *p* < 0.025), but not statistically significantly higher than the LP one (M = 3.42, SD = ±2.06; *p* > 0.05). There was a statistically significant difference between ratings participants gave for perceptual match to the three photographs as determined by one-way ANOVA: F (2,50) = 6.153, *p* < 0.025. Post hoc tests using the Bonferroni correction revealed that the mean ratings obtained by the NP (M = 3.61, SD = ±1.72) were statistically significantly higher compared to the ones obtained by the FP (M = 2.70, SD = ±1.44; *p* < 0.05), but not statistically significantly higher than the LP one (M = 2.35, SD = ±1.72; *p* > 0.05).

**Figure 8:**
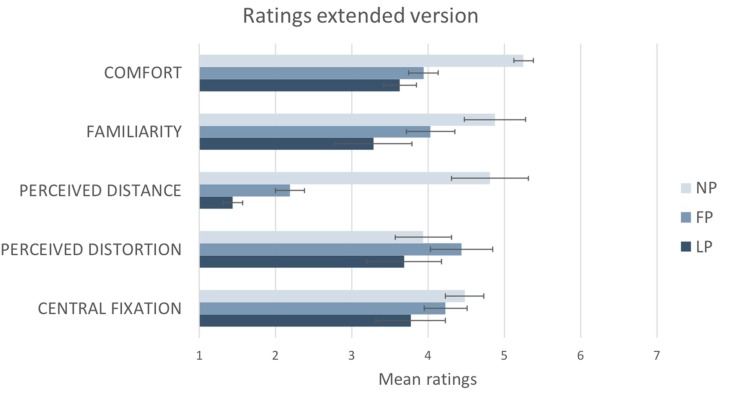
The graph reports the average ratings from the extended version of the questionnaire (*N* = 16) on a 7-point Likert scale for each photograph: Natural (NP), Fisheye (FP) and Linear (LP). Standard errors are represented in the figure by the error bars attached to each column.

Results from the sixteen participants that completed the extended version are summarized in Figure 8. There was a statistically significant difference between comfort ratings participants gave to the three photographs as determined by one-way ANOVA: F (2,30) = 6.349, *p* < 0.025. Post hoc tests using the Bonferroni correction revealed that the mean ratings obtained by the NP (M = 5.25, SD = ±1) were statistically significantly higher compared to the ones obtained by FP (M = 3.94, SD = ±1.1; *p* < 0.025) and LP (M = 3.63, SD = ±1.8; *p* < 0.05).

There was a statistically significant difference between familiarity ratings participants gave to the three photographs as determined by one-way ANOVA: F (2,30) = 6.104, *p* < 0.025. Post hoc tests using the Bonferroni correction revealed that the mean ratings obtained by the NP (M = 4.9, SD = ±1.5) were statistically significantly higher compared to the ones obtained by the LP (M = 3.2, SD = ±1.9; *p* < 0.05), but not statistically significantly higher than the FP one (M = 4.0, SD = ±1.6; *p* > 0.05).

There was a statistically significant difference between perceived distance ratings participants gave to the three photographs as determined by one-way ANOVA: F (2,18.343) = 35.195 *p* < 0.001. Post hoc tests using the Bonferroni correction revealed that the mean ratings obtained both by NP (M = 4.81, SD = ±2) and FP (M = 2.19, SD = ±0.750) were statistically significantly higher compared to the ones obtained by the LP (M = 1.44, SD = ±0.512; *p* < 0.025), but also that NP ratings were statistically significantly higher than FP (*p* < 0.001).

Results did not show any statistically significant difference between perceived distortion ratings participants gave to the three photographs (*p* > 0.05). There was a statistically significant difference between central fixation ratings participants gave to the three photographs as determined by one-way ANOVA: F (2,30) = 4.563 *p* < 0.025. Post hoc tests using the Bonferroni correction revealed that only the mean ratings obtained by NP (M = 4.47, SD = ±0.52) were statistically significantly higher compared to the ones obtained by the LP (M = 3.77, SD = ±0.87; *p* < 0.025). No other significant difference was found.

## 5. Discussion

Our study investigated the way visual space has been depicted by artists, and how artistic depictions of wide-angle scenes are rated compared to standard wide-angle photographs. We used a unique method of mapping visual space, based on an artistic rendering of the binocular visual field with a given fixation point. This method may have advantages for those seeking to establish a global metric for visual space [1,3] over other previous methods in that it treats visual space holistically by including wide eccentricities and binocularity. Experiment 1—online comparison—showed that participants expressed a clear preference for the natural perspective photograph (NP) compared to two other common alternate image geometries, namely fisheye photograph (FP) and linear photograph (LP). Experiment 2—real space comparison—confirmed previous findings using a different experimental setting, where participants could compare the three different photographs with the real scene. In the real space condition, the NP was significantly preferred, judged as having significantly higher spatial presence and being significantly more comfortable to look at compared to FP and LP. Participants rated NP as significantly higher than LP for ecological validity and perceptual match, but no significant difference was found between NP and FP ratings. We suggest that the characteristics of the NP pictorial space compensated for the previously discussed limitations of standard curvilinear and linear geometrically-accurate photographs, especially under natural viewing conditions which are at a comfortable distance (60 cm) from a standard sized display. Moreover, when directly comparing the perceived distance of the object in the photographs to the object in the real scene NP reported significantly higher scores compared to FP and LP, with FP being also significantly higher than LP. These results further confirmed that NP was significantly the most effective in conveying a realistic representation of object size in the depicted space compared to both the LP and the FP ones. Considering its widespread use in imaging technology, we might have expected that LP would have been rated as more familiar to participants compared to FP and NP. However, our results showed somewhat surprisingly that NP was rated as being significantly more familiar and easier to fixate in the center of the image compared to the LP, but not significantly higher than the FP. When directly asked to rate the perceived distortion in the image (“When looking at the central ball the image seems very distorted”) participants did not report any significant difference between the three geometries: this was the only measure in which we found no significant difference between the three geometries. This might be because LP has the advantage that, of the three photographs, it preserves the highest degree of linearity of the real 3D grid room on all axes, which would have been evident to the participants in Experiment 2. However, it is interesting to note that the image that preserved most the objectively straight lines in the 3D grid room obtained the lowest ratings on all the psychological variables we measured compared to the two images showing the same lines as highly curved. Overall, we found supporting evidence for our hypothesis that people prefer the NP to the more common wide-angle photographs because they perceive it as being closer to the experience of viewing a real scene.

A possible explanation for our results might be the link with the well-known preference for curvature, a phenomenon widely investigated in psychology and the subject of much recent experimental work [82,83]. It goes beyond the scope of this article to fully discuss the issue in this field, but it is worth noting the two main hypotheses advanced to explain this phenomenon: (1) that preference for curvature is a result of disliking for sharpness; (2) that curved forms are easier for the human visual system to process and therefore more comfortable to view (for a review on this issue see [84]). Penacchio and Wilkins [85] found that curvilinear images are more comfortable to look at compared to pictures with sharp or linear shapes and Le et al. [86] found the same results both when they measured visual discomfort in laboratory and real space conditions, looking directly at urban scenes. We suggest that NP may have a more natural distribution of curvature compared to the other perspectives. It is also possible that the preference for the NP photograph and the higher comfort associated with it might have influenced familiarity judgments, even though participants would have been exposed significantly more to the linear perspective photographs in their everyday life considering its extensive use in photography, cinematography, and computer graphics [80]. These results are surprising considering that, as also discussed in the introduction, linear perspective has been argued by many eminent theorists to be the only accurate way to depict visual space. It is also interesting to note that a study by Erkelens [87] found a tendency among many artists trained in linear perspective to depict equidistant spatial intervals non-veridically, but in a way that nevertheless appears natural in the paintings. Our results also support the hypothesis that the NP records key elements of human visual perception, presents a more ecologically valid representation of human field of view, and a more realistic representation of object relative-sizes [88,89].

There are limitations of the present study that may constrain the extent to which we can generalize from these results. First, the drawing made of the 3D grid room and the three photographs used as stimuli all contain a significant amount of approximation. The experience of visual space is inevitably complex and subjective, and determining precise values for all its features may not be possible. The drawing process is itself subjective and cannot be relied on to provide absolute consistency or accuracy. A key finding of the preparatory research for this study was that it is not possible to precisely replicate the shape or contents of the artistically generated natural perspective by computationally modifying standard photographs. Drawings are not a projection in the strict mathematical or optical sense but a composite of a partly imagined visual space based on prior knowledge of the space, memory, estimated peripheral form, and colored by artistic judgment. Moreover, the computational conversion process also required further creative judgments to best match both the initial drawing and the subjective experience of the visual space itself. Therefore, we must accept that the many interconnected image making parameters that comprise the photographic stimuli in these experiments could not be precisely controlled simultaneously, such as overall geometric form, the field of view, aspect ratio, consistent resolution across all images, and the shape of the image borders. We should also note that the NP stimulus was generated based on perceptual and creative judgments made by two artists only. As a follow-up to the present study, we will gather a larger sample of reports from a more diverse participant pool using a tool based on a computer-generated 3D model of a real scene that can be adjusted incrementally to match the structure of the scene as perceived by the participants. Part of the challenge of creating such an experimental tool is the need to be able to modify the geometry of the 3D model non-linearly, and this has entailed developing a new form of 3D graphics engine based on novel non-linear projection techniques that involve modifying Z-depth values as well as X and Y values [70]. This contributes to long-standing work in computer graphics aimed at improving the perceptual appearance of wide angle 2D and 3D images by using non-linear projections [90,91,92,93,94,95]. Future research might include testing whether the NP stimuli are faster and easier to process than standard classes of geometrically-accurate photographs, manipulating perceptual fluency and exposure time, since these have been shown to influence familiarity and emotional valence of stimuli [96,97].

We believe there may be potential practical applications for the present research. To depict visual space, we often rely on imaging technologies such as cameras and computer graphics engines. However, these devices still fundamentally rely on the optical principles of linear perspective [80]. As a result, they suffer the same limitations when capturing what we see that Leonardo da Vinci recognized [98]. A standard 50 mm lens on a camera with a 35 mm sensor will capture around only 43 degrees of the horizontal visual field [99]. This is less than one third of the 180 or so degrees typically visible to humans [17]. Different methods can be used to increase the angle of view, even up to 180 degree, but they have drawbacks. A fisheye lens, for example, provides extremely wide angles of view, but can significantly minify the perceived size of objects in the center of the image (see Figure 2). Panoramic cameras or multiple stitched photographs create images with very elongated aspect ratios [80], and every wide-angle panoramic photograph will introduce varying degrees and types of curvature across the image. Further limitations include the fact that we cannot represent the first-person point-of-view in a perceptually natural way using standard linear perspective, especially since this is most often visible in the far periphery of the visual field [19]. Many of us will have had the experience of seeing an impressive building or mountain, only to be disappointed by how small and insignificant it looks in a photograph [100]. This is due to the problem noted above, that to view a linear perspective picture properly we need to locate our eye at the exact point of projection in front of the image [20,22]. Finally, linear perspective images lack binocular information because they are generally projections from a single viewing point. Most humans enjoy a “cyclopean” experience of vision, in which information from both eyes is fused into the appearance of a single image [62,67]. This cyclopean image, however, contains binocular information, such as retinal disparity, which is important to spatial and depth judgments. Stereoscopic images can depict binocular information, but special viewing devices are required to see the effect [67]. The method of depicting visual space described here using both artistic and technological techniques may usefully contribute to improving the perceived naturalism of imaging technologies.

## 6. Conclusions

Artists and architects first discovered the principles of linear perspective, and for hundreds of years were rigorously trained in them. Yet despite the simplicity and power of these geometrical principles and the guarantee of objective fidelity they offer for depicting three-dimensional space, artists hardly ever rigorously applied them due to their limitations. Instead, they often seemed to have favored various forms of natural perspective that tend to have more curvature, especially in the periphery, and tend to enlarge areas of scene under central fixation. When these natural perspective principles are applied to create a photograph of a wide-angle scene, people report that they prefer it, finding it as having more spatial presence, as being more comfortable to look at, and better at representing perceived distance of depicted object in the center of the image compared to images created using familiar and common perspectives, especially linear perspective. We conclude that in comfortable viewing conditions a natural perspective image is more effective at depicting wide-angle scenes than the more common perspective alternatives, despite or perhaps even because of the additional curvature they introduce. We further suggest that such natural perspectives might more closely represent the structure of human visual space than standard perspectives under these viewing conditions.

## Figures and Tables

**Table 1:** For each of the three photographs (Natural, Fisheye and Linear), the table shows: in the first raw the absolute percentage of respondents that selected each answer choice; and in the second raw the total frequencies reported for each answer choice. The total responses column reports the total answers collected for each answer choice. The ranking score column lists for each photograph the Total Index of Preference, calculated by summing the weighted ranking for each answer choice.

	1st Position	2nd Position	3rd Position	Total	Ranking Score
**Natural**	43.24%	31.08%	25.68%		
	32	23	19	74	**2.18**
**Fisheye**	36.49%	39.19%	24.32%		
	27	29	18	74	**2.12**
**Linear**	20.27%	29.73%	50.00%		
	15	22	37	74	**1.70**

**Table 2:** The table lists all items used for the questionnaire in Experiment 2. The second and the third row report items selected from the * SOPI inventory; the fourth row reports the customized item.

Psychological Variables	Items Used in Experiment 2
Liking	*“I like this picture.”*
Ecological Validity *	*“I feel that the displayed environment is part of the real world.”*
Spatial Presence *	*“I have a sense of being in the scene displayed.”*
	*“I feel like I can reach and touch the things in the displayed environment.”*
Perceptual match (customized item)	*“I think that the image represents exactly how I perceive the environment.”*

**Table 3:** The table lists all the additional items used in the extended version of the questionnaire in Experiment 2.

Psychological Variables	Additional Items Used in Experiment 2
Comfort	*“I find this picture comfortable to look at.”*
Familiarity	*“I have seen images that use this perspective often.”*
	*“Images that use this perspective are familiar to me.”*
Perceived distance	*“The ball in the image appears to be the same distance away from me as the ball in reality.”*
Perceived distortion	*“When looking at the central ball the image seems very distorted.”*
Central fixation	*“I find it easy to focus on the ball.”*
	*“I find my attention wandering away from the ball” (reverse item)*
	*“The edges of the image are distracting my focus from the central ball.”*
